# The Role of Nano-Fertilizers in Sustainable Agriculture: Boosting Crop Yields and Enhancing Quality

**DOI:** 10.3390/plants14040554

**Published:** 2025-02-11

**Authors:** Mcholomah Annalisa Kekeli, Quanlong Wang, Yukui Rui

**Affiliations:** 1 Beijing Key Laboratory of Farmland Soil Pollution Prevention and Remediation, College of Resources and Environmental Sciences, China Agricultural University, Beijing 100193, China; annalisamcharthyholomah@gmail.com (M.A.K.); wql199602@163.com (Q.W.); 2 Faculty of Resources and Environment, China Agricultural University, Shanghe County Baiqiao Town Science and Technology Courtyard, Jinan 250100, China

**Keywords:** nano-fertilizer, conventional fertilizers, application methods, yield enhancement, quality parameters

## Abstract

With the rising need for sustainable agricultural practices, nano-fertilizers have emerged as an innovative alternative to traditional fertilizers. These advanced fertilizers enhance nutrient use efficiency, promote crop growth, and minimize environmental harm by enabling precise nutrient delivery. This review evaluates various nano-fertilizer application techniques and their influence on plant growth, yield, and quality. Additionally, it explores their interactions with soil composition and microbial communities, emphasizing their role in enzymatic activity and nutrient cycling. While nano-fertilizers offer significant benefits, challenges such as proper dosage regulation, potential toxicity, and long-term ecological effects necessitate further research. This study highlights recent advancements in nano-fertilizer technology and underscores the importance of an integrated approach to optimize agricultural productivity while preserving soil health and environmental sustainability.

## 1. Introduction

In recent years, the global agricultural sector has faced increasing challenges in enhancing crop productivity while promoting environmental sustainability. Although traditional fertilizers are effective, they often result in significant nutrient losses through leaching, volatilization, and fixation. These losses decrease nutrient utilization and contribute to environmental challenges [1]. The introduction of nano-fertilizers in agriculture offers a potential solution to these challenges.

Nano-fertilizers are nutrient formulations encapsulated or coated in nanomaterials, enabling controlled nutrient release and gradual dispersion in the soil [2]. Compared to traditional fertilizer options, nano-fertilizers provide several advantages, including improved nutrient utilization, reduced environmental impact, and enhanced agricultural output and product quality [3]. This improvement is largely attributed to the nanoscale size of these fertilizers, which facilitates better absorption and penetration into plant tissues. In addition, the interaction between nano-fertilizers and soil microorganisms is a critical area of interest because soil microbes are essential organisms for soil nutrient cycling and plant growth [3]. Optimizing the application methods and dosages of nano-fertilizers remains a key area of research. Various techniques, including foliar sprays and root applications, have been investigated with varying levels of success [4]. The timing and frequency of application are also crucial factors that significantly influence crop responses and yield [5].

Concerns regarding the potential phytotoxicity and long-term impacts of nano-fertilizers on the soil ecosystem highlight the need for careful management of application rates [6]. The integration of nano-fertilizers with conventional fertilization practices has demonstrated promising outcomes across various cropping systems. Such combined strategies enhance nutrient utilization while preserving soil health [7]. This approach enables the reduction of conventional fertilizer application rates while maintaining or even improving crop yield [8]. Given the intricate interactions among nano-fertilizers, soil properties, and microbial communities, extensive research is needed to refine the application methods, types, and cropping systems. This study seeks to identify and understand the application methods of nano-fertilizers for maximizing crop yield while maintaining soil health. Additionally, it seeks to examine how soil microorganisms are affected after their interactions with nano-fertilizers, which play an important role in nutrient cycling and overall ecosystem performance.

## 2. Nano-Fertilizers: Characteristics and Advantages

Nano-fertilizers are nutrient formulations encapsulated or coated with nanomaterials that are designed to enable controlled release and gradual dispersion in the soil. These advanced fertilizers offer a slower nutrient release compared to traditional bulk fertilizers [2]. Nano-fertilizers excel in controlled and precise nutrient release, thereby improving utilization efficiency. Their nanoscale properties offer superior effectiveness in plants, especially in some studies when compared with traditional fertilizers [9].

**Figure 1:**
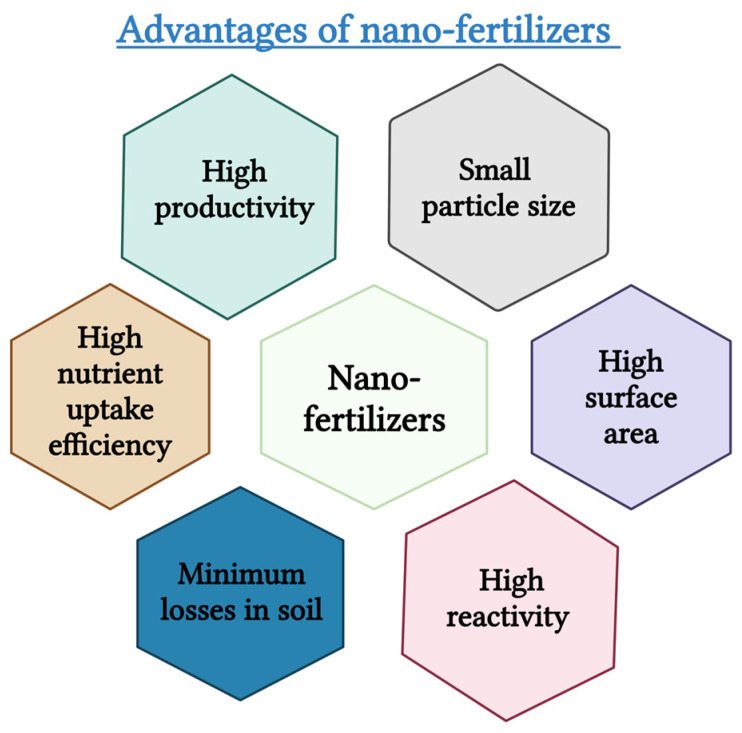
Important advantages of nano-fertilizers [22].

Nanotechnology has emerged as a transformative solution in agriculture, with nano-fertilizers playing a pivotal role. These fertilizers, which are created using nanotechnology, are nutrient carriers with sizes ranging from 1 to 100 nanometers [10]. The distinctive characteristics of nano-fertilizers, such as their nanoscale size, large surface area, and increased reactivity, render them significantly more efficient than traditional fertilizer options [11]. A key advantage of nano-fertilizers lies in their capacity to deliver nutrients in a controlled and precise manner, ensuring that all nutrients are aligned with crop requirements throughout the growing season [12]. Table 1 shows the effects of various nano-fertilizers on several crops, along with the range of doses for each crop. Research suggests that nano-fertilizers can enhance nutrient utilization by 20–30% compared to traditional fertilizers, while significantly minimizing nutrient losses to the environment [13]. Figure 1 illustrates the various advantages of nano-fertilizers, highlighting their potential to revolutionize sustainable practices.

**Table 1:** Effects of various nano-fertilizers on several crops, along with the range of doses for each crop.

Nano-Fertilizers	Range of Doses	Plant/Crop	Effects	References
Zn NPs	5–20 mg/L	*Allium cepa* L.	Reduced root growth	[14]
Nano SiO_2_ + Nano TiO_2_	100–500 ppm	*Capsicum annuum* L.	Significant increase in seed germination	[15]
ZnO NPs	100 mg/kg	*Cucumis sativus* L.	Inhibited growth	[16]
ZnO NPs	20 mg/L	*Triticum aestivum* L.	Enhanced biological yield and grain production	[17]
	10 mg/L	*Cyamopsis tetragonologa* L. *Taub*	Increased growth biological yield and nutrient contents	[18]
	10 mg/L	*Zea mays* L.	Enhanced shoot and root growth, plant height, leaf size, chlorophyll levels, and grain quality	[19]
	5–20 mg/L	*Solanum melongena* L.	Decreased germination, root length, and leaf area in culture media, but an increase in these parameters in soil conditions	[20]
Cu NPs	20–80 mg/kg	*Coriandrum sativum* L.	Reduced germination and shoot development	[21]
	50–500 mg/L	*Solanum lycopersicum* L.	Enhanced antioxidant and improved fruit firmness	[22]
	10–20 mg/L	*Lactuca sativa* L.	Reduced seed growth and dry weight and impacted water balance and nutrient content	[23]
	130–660 mg/kg	*Lactuca sativa* L.	Enhanced root and shoot length	[24]
Nano-Fe_2_O_3_	500–1000 mg/L	*Cuminum cyminum* L.	Enhanced stem length and iron concentration	[25]
Nano-N	25–100%	*Oryza sativa* L.	Boosted the number of tillers per plant, height and dry weight	[26]
Nano-potash	1500–2500 mg/L	*Arachis hypogea* L.	Enhanced shoot length, stem biomass, biological yield, and number of flowers per plant	[27]

The environmental benefits of nano-fertilizers are particularly noteworthy. Nano-fertilizers enhance sustainable agricultural practices by optimizing nutrient utilization and reducing nutrient losses due to leaching and volatilization [28]. This is especially important given the growing concerns about environmental pollution and soil degradation associated with the use of conventional fertilizers [29].

Recent advances in nano-fertilizer technology in agriculture have led to the development of various formulations, including nano-urea, nano-phosphorus, and nano-micronutrients [7]. These innovations have shown promising results in both controlled environments and field conditions. For example, nano-Zn and nano-Fe fertilizers significantly increased crop yields and promoted nutrient absorption [30]. The integration of nano-fertilizers with other agricultural technologies has also shown synergistic effects. Despite these advantages, several challenges remain in the widespread adoption of nano-fertilizers. These challenges include the need for standardization of production methods, assessment of long-term environmental impacts, and cost-effectiveness considerations [2].

## 3. Application Methods and Dosage Optimization of Nano-Fertilizers

**Figure 2:**
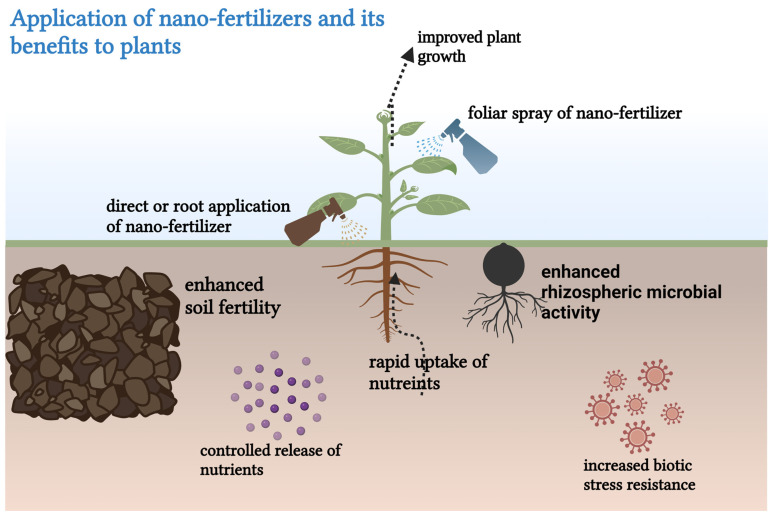
Application of nano-fertilizers and their benefits to plants, including enhanced rhizosphere microbial activities, enhanced soil fertility, rapid uptake of nutrients, controlled release of nutrients, and increased biotic stress resistance [31].

The efficiency of nano-fertilizers is highly influenced by their application methods, which depend on factors such as their nanoscale properties, soil composition, environmental conditions, delivery systems, and plant species [31]. Studies have explored various application techniques, including foliar spraying, soil or root application, and combined methods, across a range of crop types [32]. Figure 2 below shows the different application methods of nano fertilizers and their benefits to plants. 

Studies have revealed that nano-fertilizers could enhance agronomic yield by 10–80% compared to conventional fertilizer options at a reduced application rate [10]. The dosage optimization of nano-fertilizers is extremely important because it will determine both economic and environmental outcomes. Adjusting dosages has been demonstrated to significantly enhance nutrient uptake efficiency and boost crop productivity [8]. For example, results from experiments on maize cultivation revealed that the optimized uptake of conventional fertilizers, in combination with nano-fertilizers, achieved higher yields compared to the traditional approach while minimizing the total amount of fertilizer used [33].

However, applying nano-fertilizers comes with challenges that require careful consideration. Assessing the potential toxicity of nanomaterials and their long-term impacts on the soil ecosystem is essential [34]. Also, a critical research area remains the standardization of application methods and dosage optimization, which can vary according to crops and environmental conditions [35].

These methods and dosages of nano-fertilizer applications can be optimal at different levels depending on the crops, their growth stages, environmental applications, soil proxy, and various characteristics [36]. Studies in recent years have shown that the timing and method of nano-fertilizer application might be crucial for achieving a better response [37]. Today, as sustainable agriculture is gaining ground, the proper application and dosage of nano-fertilizers are critical for their effective use in overcoming the present challenges in the agricultural field.

### 3.1. Foliar Application

The foliar application of nutrients represents a targeted approach in which fertilizers are directly applied to plant leaves, enabling rapid absorption and translocation of nutrients throughout the plant system [38]. This approach has attracted significant interest because of its capability to overcome limitations associated with soil-applied fertilizers, such as nutrient fixation, leaching losses, and poor soil conditions that may impede nutrient uptake [39]. One example of this application method is the study on *Cucurbitta pepo* (*pumpkin*), where it was observed that the fertilizer was absorbed rapidly and there was an acceleration in the transition of elements when the fertilizers were foliar sprayed; this resulted in the demonstration of enhanced drought tolerance by enhancing the plant’s response and water retention capabilities [40]. Foliar spraying of nano-fertilizers has been found to reduce fertilizer usage or even increase crop yields [41].

In addition, combining nano-fertilizers with traditional fertilization programs has shown promising outcomes across various crops. An example is the study on maize, which revealed that the foliar application of nano-NPK fertilizer, alongside reduced amounts of conventional fertilizers, improved the growth of maize, yield components (ear grain weight, cob weight, 500-grain weight, and number of grains per ear), and nutrient utilization [33]. Positive results like these have also been observed in rice [42] and wheat cultivation systems. In an experiment by Al-Juthery et al. [43], the study focused on the application of various nano-fertilizer treatments through foliar spraying on wheat. At the end of the study, it was identified that foliar application of nano-fertilizers facilitates rapid absorption, enhancing fertilizer efficiency, crop growth, and nutrient uptake. The findings highlighted that combining different nano-fertilizer formulations, such as N + P + K, N + P, N + K, P + K, and conventional NPK + TE with foliar application significantly boosted wheat yield. This improvement was attributed to enhanced root growth and vegetative development, which promoted the uptake of macro- and micronutrients. Another study was conducted by M.M. El-Azeim [44], which compared the effects of the foliar application of NPK nano-fertilizers to those of traditional chemical fertilizers. The results indicated that applying NPK nano-fertilizers at rates equal to or below the recommended levels for conventional NPK fertilizers had a positive impact on biological yield, economic yield, and the fresh and dry weights of tubers and vegetative parts of potatoes. The foliar application of NPK nano-fertilizers on the potatoes was noted to increase plant vegetative growth and leaf area, which enhanced the utilization of solar radiation essential for the photosynthesis process and chlorophyll formation. This led to the vigorous growth of the potato, hence the increased fresh and dry weight noted after the experiment. Foliar spraying, in particular, significantly enhanced potato production, improving both yield and quality for both nano- and conventional fertilizers. Remarkably, under field conditions, the foliar application of NPK nano-fertilizers at 25% or 50% of the recommended rate was found to be equally or even more effective than applying 100% of conventional NPK fertilizers in improving potato yield-associated parameters [44].

The timing and frequency of foliar applications also play crucial roles in determining their effectiveness. Research has demonstrated that the timing of application is crucial for optimizing nutrient uptake and efficiency [30]. Applying nano-fertilizers via foliar spraying during the vegetative and reproductive stages significantly improved yield characteristics and overall crop performance [45]. The vegetative stage of a plant is a stage where the plant is focused on growing roots, leaves, and other structures that are essential for supporting future reproductive processes; nano-fertilizers are known to be more readily absorbed by the plant’s surface due to their small size and high surface area, which allows for a better uptake of nutrients. Hence, when nano-fertilizers are foliar sprayed on plants during their vegetative stage, this can lead to robust growth, healthier foliage, and stronger stems, setting a solid foundation for the growth of the plant. The vegetative stage, which includes flowering, fruiting, and seed formation, is the most crucial stage for determining yield. Foliar application at this stage provides proper nutrition to plants, which can enhance flower formation, pollen viability, and pollination success and then increases the yield of the plant. The results of the application of Formax nano-fertilizers at a concentration of 5 mL/L, administered twice as a foliar spray during the vegetative growth stage, demonstrated a superior ability to enhance the height of coriander, which led to more branching and a full umbrella capacity due to the presence of essential nutrients and improved use of environmental factors, resulting in a higher yield [46].

In spite of these merits, many of the facets of foliar nano-fertilizer application need more scrutiny. Concerns regarding the environmental footprint of conventional fertilizers have also spurred interest in foliar applications of nano-fertilizers. The foliar application of nano-fertilizers offers a sustainable approach to overcoming nutrient loss and unbalanced fertilization due to the low dosage and targeted application mechanisms [35]. There remains a need to further understand, for example, the mechanisms of nutrient absorption through leaf surfaces, the optimal particle size for maximum sorption, and the interaction with potential plant defense systems to enhance or suppress fungal growth [47]. Moreover, the harmonization of application protocols for different crops and agronomic conditions is still a struggle [47]. The economic potential of the applications of foliar nano-fertilizer also needs to be considered. Although the upfront costs of nano-fertilizers may still be higher compared with conventional fertilizers, lower application rates and better nutrient utilization help to balance this cost [48].

### 3.2. Root Application

Nanoparticles for fertilizer application directly to the roots offer a way to localize nutrient delivery, reducing nutrient losses and maximizing plant uptake. Nanoscale fertilizers enable nanoparticles to go through the epidermis, endodermis, and xylem after application to the root and then be sent to the plant’s aerial parts. Nanoparticles pass through gaps in the cell wall that range from 3 to 8 nm [49]. Studies have suggested that nanoparticles might enter into plants through root tips or the sections devoted to lateral roots (where the Casparian strip has holes).

For nanoparticles to accumulate within the epidermal layers of roots, they must pass through the cell walls and plasma membrane. Typically, cell wall pores range in size from 3 to 8 nanometers [50], which is too small for nanoparticles to pass through. Nevertheless, research has revealed that nanoparticles can be ingested since large holes are created in the cell walls [51]. Certain nano-fertilizers applied to roots have also been shown to enhance beneficial microbial activity in the rhizosphere, according to some studies [52]. One of the benefits of applying nano-fertilizers to the roots is that they facilitate nutrient absorption, promote the growth of mycorrhizal fungi, and enhance nitrogen fixation by utilizing nitrogen-fixing bacteria. As soil serves as the primary medium for applying nano-fertilizers, it is essential to understand their effects on soil- and root-associated microbial communities [31].

**Figure 3:**
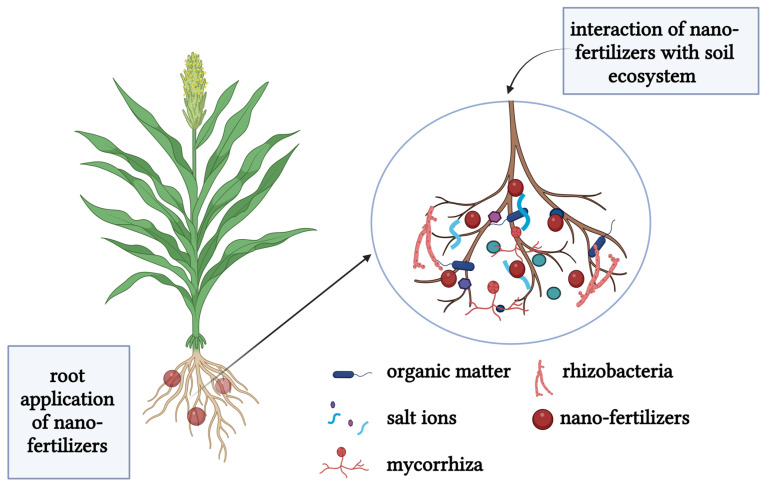
Interaction of nanofertilizers with soil ecosystem [55].

When nano-fertilizers are applied to the roots of plans, they engage with bacteria, fungi, and other microorganisms that establish beneficial symbiotic relationships with plants. They may also interact with compounds such as humic and organic matter as shown in Figure 3 below, which can enhance their bioavailability in the rhizosphere and facilitate plant absorption and uptake [53]. An example is when humic acids and organic matter in soil self-assemble into aggregates to enhance stability and subsequently enhance their bioavailability with nanomaterials [54].

#### 3.2.1. Effect of Nano-Fertilizers on Soil- and Plant-Associated Microorganisms

Soil properties play a crucial role in affecting the dispersion, aggregation, stability, immobilization, bioavailability, and transport of nanoparticles [56]. Some research has suggested that the effectiveness of nanoparticles depends solely on their application method and concentration in terms of determining their impact on soil properties. For example, a study on titanium oxide nanoparticles at concentrations ranging from 1 to 100 mg/kg found no toxic effects on soil microbial communities; however, in contrast, copper oxide, zinc oxide, and silver nanoparticles demonstrated toxicity to soil microorganisms at comparable concentrations [57].

Recent research has shown that applying nano-fertilizers directly to the roots is crucial in the interaction between the soil ecosystem. A study revealed that the direct or root application to soil of metallic silver nano-fertilizer increased the abundance of protobacteria and acidobacteria and also improved microbial metabolic activity [58]. In another study, the same metallic silver nano-fertilizer was directly applied to the soil, and it increased the protobacteria abundance, decreased acidobacteria abundance, and increased the degradation potential for soil pollutants like xenobic compounds, which indicated that the microbial community was more efficient at breaking down synthetic or harmful chemicals that entered into the environment, including pesticides, herbicides, and industrial pollutants that came into contact with the soil [59]. Such nano-fertilizers, in addition to improving the nutrient cycle, also improve soil health, leading to such effects.

McGee et al. [59] investigated the impacts of aluminum oxide and silicone oxide nanoparticles applied at a concentration of 50 mg/kg on bacterial biodiversity. The presence of both of the nanomaterials used was shown to be non-toxic to most organisms at the concentrations used in the study. Aluminum oxide and silicon oxide nanoparticles were found to have little effect on bacterial diversity, unless they were concentrated in large quantities in the soil.

#### 3.2.2. Soil pH

Soil pH is another important factor to consider when evaluating the effects of nanomaterials on the ecosystem. After the application of nanomaterials through the soil, a decrease in the pH was seen due to the nature of nanomaterials applied, which increased the accessibility of nutrients for the plant; however, a decrease in pH (acidic soils) also facilitated dissolution, potentially leading to the generation of harmful reactive oxygen species depending on the concentration of nano-fertilizers and the overall soil conditions. Reactive oxygen species can contribute to both plant and microbial defense mechanisms and facilitate the degradation of pollutants through the decomposition of toxic compounds; furthermore, it triggers stress responses in the plant body. Additionally, nano-fertilizers helps inhibit their use due to soil particles binding them if they have a great adsorption capacity, such as in the case of phosphorus in acidic soils [60].

Sulfur is known to have acidifying properties on soils; thus, when sulfur is applied in high-pH conditions, it gradually oxidizes to form sulfuric acid, which separates in water to produce hydrogen ions that lower the soil pH [61]. A study by Esmaeli et al. [62] also obtained results suggesting that applying nano-sulfur to soil reduced the soil pH. The decreased soil reaction pH was found to be a result of the microbial oxidization of sulfur to sulfuric acid. After the experiment was conducted, it was identified that compared to the mineral sulfur used in the experiment, nano-sulfur was better due to the unique properties of nanomaterials, such as the high surface-to-volume ratio and many other properties of nanomaterials.

Studies have found that zinc oxide nanoparticles have an increased positive effect on the bacterial population in saline alkali soil than that in weakly acidic soil. This difference was attributed to greater microbial diversity in saline alkali soil. Acidic soil was reported to be more negatively impacted by ZnO nanoparticles than calcareous soil with respect to soil microbial enzyme activities [63]. However, despite a high metal sorption capacity, calcareous soil was more negatively influenced than acidic soil in terms of microbial catabolism [64].

Thus, in conclusion, nanomaterials have a positive effect on soil properties such as pH; however, the effect depends on the type of nanomaterial applied, the concentration of the nanomaterial, and the soil environment itself.

## 4. Impact on Crop Yield and Quality Parameters

### 4.1. Crop Yield

With the global increase in population, there is a need for good sustainable agricultural practices that can increase crop yield and reduce adverse effects on the environment. While traditional fertilizers boost crop yields, their use comes with drawbacks, such as the negative impact on the soil, emissions of greenhouse gases into the atmosphere, and eutrophication caused by the runoff of traditional fertilizers into water bodies [65]. The problems these limitations create not only diminish traditional fertilizers’ efficiency but they also result in environmental pollution, soil degradation, and economic losses, and they are therefore unsustainable.

Field and greenhouse experiments have demonstrated that using different nano-fertilizers can improve yields. Specifically, applying NPK nano-fertilizers has been effective in balancing rice yield and quality while improving fertilizer efficiency [66]. Kumar et al. [7] stated that during tillering stages, the foliar spraying of nano-nitrogen and nano-copper fertilizers led to increased grain and straw yields in wheat. Additionally, nano-fertilizers enhance nutrient bioavailability, stimulate various plant pathways and enzymes, boost root biomass, and boost the microbial population in the rhizosphere, which are key factors for optimizing crop productivity. In a greenhouse experiment, Ghahremani et al. [67] investigated the effects of varying concentrations of nano-calcium and nano-potassium chelate fertilizers on the quantity and quality traits of basil. It was effective in improving the 1000-seed weight, harvest index, grain yield, and biological yield compared to the control and other treatments. Asadzade et al. [68] investigated the impact of traditional fertilizers and nano-fertilizers (ZnO and SiO_2_) on sunflower yield and harvest index. The study found that ZnO nano-fertilizer significantly enhanced parameters such as head diameter, seed yield, 1000-seed weight, and the number of seeds per head compared to the control plot and other treatments. Manikandan and Subramanian [69] carried out two greenhouse experiments utilizing soils with different textures—Inceptisols and Alfisols—to assess the growth-promoting effect of zeolite-based nitrogen nano-fertilizers in comparison to conventional fertilizers. The findings revealed that maize grain yield was significantly greater with the nano-zeolite treatment than with the conventional urea treatment used as the control. Abd EL-Azeim et al. conducted a study to evaluate the effects of soil-applied NPK nano-fertilizers and chemical fertilizers on potato crop yield and its parameters. After the investigation, it was found that foliar application significantly influenced the effectiveness of both nano- and conventional fertilizers on potato production and yield quality compared to soil application. All treatments involving nano- or chemical fertilizers improved potato yield parameters, including the fresh and dry weights of tubers, relative to the control. These findings suggested that prioritizing the use of nano-fertilizers was advisable, but at equal or lower application rates, to achieve optimal potato yield.

In sustainable agriculture, nano-fertilizers can be used for more than yield enhancement. As advanced fertilizers, they are expected to increase nutrient utilization, decrease environmental pollution, and improve crop quality [70]. The responsive behavior of nano-fertilizers has established their potential to reduce the total fertilizer requirement without compromising or even increasing crop productivity [71]. The development of crop-specific fertilizer management strategies has gained increasing attention. Different crops exhibit varying nutrient requirements and absorption patterns, necessitating tailored approaches to fertilizer application [72]. Understanding these specific needs helps in developing more efficient and sustainable fertilization practices. Recent studies have also highlighted the importance of soil type and environmental conditions in determining the effectiveness of nano-fertilizers.

The interaction between soil properties and nano-fertilizer efficiency has been shown to significantly influence crop response and yield outcomes [73]. The overall crop response to nano-fertilizer application in the soil depends solely on how the physical, chemical, and biological properties interact. The pH, texture, organic matter, salinity, and nutrient status of the soil can all impact how effective the nano-fertilizers used will be absorbed and how well plants will utilize the nutrients administered. In soil with favorable conditions for nutrient uptake and retention, there will be a notable increase in crop growth, yield, and overall performance. This understanding is crucial for developing region-specific fertilizer recommendations.

### 4.2. Quality Parameters

Nano-fertilizers have gained attention for their ability to enhance the quality of agricultural products by improving nutrient composition, biochemical properties, and post-harvest characteristics. The ability of these nano-fertilizers to enhance nutrient adsorption and optimize plant metabolic functions contributes to improving crop quality.

Recent research has demonstrated significant improvements in the nutritional profile by increasing the uptake of essential nutrients, including nitrogen, phosphorus, potassium, and micronutrients, due to nano-fertilizer applications. A study on rice cultivation showed that the foliar application of nano-formulated DAP significantly increased the concentrations of nitrogen phosphorus and potassium in grains, which enhanced the grain protein content, amino acid composition, and overall nutrient efficiency [74]. Qiang et al. [75] conducted a study to assess the effectiveness of a slow-release nano-fertilizer on winter wheat and summer corn quality. The findings revealed that applying these nano-fertilizers significantly enhanced protein content.

Beyond nutrient enhancement, nano-fertilizers have been found to influence plant biochemical and physiological processes. The study by M.M. Al-Azeim et al. [44] evaluated the nutrient utilization of NPK. The findings showed that all treatments significantly enhanced nutrient use efficiency per hectare across all application rates. The large surface area and small particle size of nano-fertilizers, which is smaller than the pore size of potato leaves, allowed for improved penetration into plants vegetative tissues. This enhancement facilitated the better uptake and utilization of NPK nano-fertilizers compared to the conventional chemical fertilizer used in the study. Consequently, the treatment in the study, which contained 50% NPK nano-fertilizer, demonstrated the highest efficiency for nitrogen, phosphorus, and potassium.

Jian et al. [76] achieved findings that revealed that applying nano-synergistic fertilizers led to a 7.52% decrease in protein content, while fat content increased significantly by 33% in winter wheat, suggesting a shift in the plants’ metabolic pathways, altering nutrient processing, energy utilization, or biochemical compound synthesis. Similarly, Khater [77] found that the application of titanium dioxide nanoparticles (TiO_2_ NPs) boosted chlorophyll production, enhancing the contents of amino acids, total sugars, total phenols, total indoles, and pigments in coriander, all of which are important indicators of improved crop quality. Sharifi et al. [78] reported that nano-iron application had a notable impact on the growth and quality of corn, which is a key forage crop. Iron nanoparticles were found to improve total dry mass, crude protein levels, soluble carbohydrates, and phosphorus concentrations in the crop. Furthermore, both nano- and chemical zinc formulations resulted in a significant increase in the leaf chlorophyll index, plant height, total dry biomass, crude protein, and soluble carbohydrate levels when compared to the control plants.

The smaller particle size enhances the specific surface area and number of particles per unit area, enabling nano-fertilizers to interact more efficiently with nutrients. This improved interaction, which facilitates better nutrient penetration and absorption, ultimately increases nutrient utilization efficiency, which plays a crucial role in optimizing crop quality and overall productivity [79].

## 5. Environmental Considerations

The global agricultural industry faces significant challenges in meeting growing food demand while ensuring environmental sustainability. With the global population expected to surpass 9 billion by 2050, it is essential to enhance agricultural productivity while minimizing environmental impact [80]. Although traditional fertilizers play a key role in crop production, they often exhibit low nutrient utilization efficiency and contribute to problems such as soil degradation, water contamination, and greenhouse gas emissions [81]. In recent years, nanotechnology has emerged as a promising approach to addressing these challenges in agriculture. Nano-fertilizers, which are either scaled down to the nano scale or encapsulated in nanomaterials, have demonstrated significant potential to revolutionize agricultural practices by enhancing nutrient use efficiency and mitigating the use of environmental fertilizers [82]. These innovative materials provide several benefits over conventional fertilizers, including controlled nutrient release, improved plant uptake, and reduced nutrient loss [13]. The advancement of nano-fertilizers marks a major breakthrough in agriculture. These fertilizers can be engineered to synchronize nutrient release with crop requirements, thereby potentially lowering fertilizer usage while sustaining or enhancing crop yields [83]. Research has shown that nano-fertilizers can boost nutrient absorption efficiency by 20–30% compared to conventional fertilizers, resulting in improved crop performance [10].

However, the adoption of nano-fertilizers in agriculture requires the careful consideration of various environmental factors. Although nanomaterials possess unique properties that provide significant advantages for agriculture applications, they also raise concerns regarding potential environmental impacts [84]. Their small size and high reactivity may lead to interactions with soil ecosystems that are not yet fully understood, potentially affecting non-target organisms [85].

Understanding the environmental dynamics and interactions of nano-fertilizers in agricultural systems is crucial. Research has shown that different nano-fertilizer formulations release nutrients and interact with the environment in diverse ways [86]. For example, studies involving graphite nanoparticles combined with conventional fertilizers have shown a reduction in nutrient leaching [87]. The development of environmentally friendly nano-fertilizers has become a key focus of agricultural research, with advances in green synthesis techniques and the use of biodegradable materials offering the potential for more sustainable formulations [88]. These strategies focus on preserving the advantages of nano-fertilizers while reducing environmental risks. Measures to ensure their safe use include creating risk assessment protocols and implementing safe-by-design approaches that assess the accumulation of nanomaterials in soil and their long-term impacts on soil health and ecosystem functionality [89,90]. Incorporating nano-fertilizers into sustainable agricultural practices necessitates balancing the demand for higher productivity with environmental conservation. Recent research has emphasized their potential to enhance crop yields while maintaining ecological safety [91].

The widespread adoption of nano-fertilizers requires ongoing research into their environmental effects and the establishment of suitable regulatory frameworks. A thorough assessment of the behavior of nano-fertilizers across different agricultural systems, as well as the potential risks to ecosystems and guidelines for safe application, are essential to ensure their role in sustainable agriculture [29].

## 6. Conclusions

Nano-fertilizers have emerged as a promising innovation in agriculture, enhancing nutrient efficiency, improving crop yields, and reducing the environmental footprint of traditional fertilization methods. This review examined various application techniques and their interaction with soil properties and microbial communities, highlighting their role in sustainable crop production. Despite their advantages, concerns regarding optimal dosage, potential toxicity, and long-term environmental effects remain. To maximize their benefits, further research is needed to refine application methods, evaluate their long-term impact, and integrate them effectively into conventional farming systems. Addressing these challenges will enable nano-fertilizers to contribute significantly to sustainable agricultural development while ensuring soil health and environmental safety.

## Data Availability

Data is contained within the article.
